# Treatment with a neutrophil elastase inhibitor and ofloxacin reduces *P. aeruginosa* burden in a mouse model of chronic suppurative otitis media

**DOI:** 10.1038/s41522-021-00200-z

**Published:** 2021-04-06

**Authors:** K. M. Khomtchouk, L. I. Joseph, B. B. Khomtchouk, A. Kouhi, S. Massa, A. Xia, I. Koliesnik, D. Pletzer, P. L. Bollyky, P. L. Santa Maria

**Affiliations:** 1 Department of Otolaryngology, Head and Neck Surgery, Stanford University, Stanford, CA USA; 2 Department of Medicine, Section of Computational Biomedicine and Biomedical Data Science, University of Chicago, Chicago, IL USA; 3 Department of Otolaryngology, Head and Neck Surgery, Tehran University of Medical Sciences, Tehran, Iran; 4 Department of Medicine, Infectious Diseases, Stanford University, Stanford, CA USA; 5 Department of Microbiology and Immunology, University of Otago, Dunedin, New Zealand

**Keywords:** Biofilms, Antimicrobials, Pathogens, Bacteria

## Abstract

Chronic suppurative otitis media (CSOM) is a widespread, debilitating problem with poorly understood immunology. Here, we assess the host response to middle ear infection over the course of a month post-infection in a mouse model of CSOM and in human subjects with the disease. Using multiparameter flow cytometry and a binomial generalized linear machine learning model, we identified Ly6G, a surface marker of mature neutrophils, as the most informative factor of host response driving disease in the CSOM mouse model. Consistent with this, neutrophils were the most abundant cell type in infected mice and Ly6G expression tracked with the course of infection. Moreover, neutrophil-specific immunomodulatory treatment using the neutrophil elastase inhibitor GW 311616A significantly reduces bacterial burden relative to ofloxacin-only treated animals in this model. The levels of dsDNA in middle ear effusion samples are elevated in both humans and mice with CSOM and decreased during treatment, suggesting that dsDNA may serve as a molecular biomarker of treatment response. Together these data strongly implicate neutrophils in the ineffective immune response to *P. aeruginosa* infection in CSOM and suggest that immunomodulatory strategies may benefit drug-tolerant infections for chronic biofilm-mediated disease.

## Introduction

*Pseudomonas aeruginosa* is a World Health Organization priority pathogen due to its ability to evolve resistance to all available antibiotics^1^. *P. aeruginosa* can cause a variety of biofilm-associated diseases including cystic fibrosis (CF) and chronic suppurative otitis media (CSOM), as well as implant-associated infections, and chronic cutaneous wound ulcers^2–7^.

Pseudomonal CSOM is the leading cause of permanent hearing loss in developing countries and accounts for the largest fraction of chronic Pseudomonal biofilm diseases, affecting over 300 million individuals worldwide^8–10^. Many patients eventually require surgery but relapse is still very common^11,12^. Traditional antimicrobial approaches fail to eradicate biofilms in CSOM and other settings due increased drug tolerance^13,14^. Fluoroquinolones remain the standard of care. However, efficacy is reduced in chronic infections compared to newly acquired infections, and rates of fluoroquinolone resistance are rising^15^.

Evolutionarily conserved defense mechanisms of bacteria include ways to subvert the host immune response—the role of neutrophils in biofilm-mediated diseases is an interesting topic of research in this regard, having both detrimental and protective roles in infection response^16^. Extracellular traps (NETs) are particularly interesting in cases of biofilm-mediated disease as NETs are primarily made of dsDNA (along with various cytosolic and granule proteins) and dsDNA is also a component of the extracellular biofilm matrix. There is evidence to suggest that necrotic neutrophils contribute DNA to the biofilm extracellular matrix as a result of failed attempts to clear infection^17,18^. Host biofilms are stronger and more developed in the host versus in a petri dish, and NETs can add to the integrity of the aggregate. It has previously been demonstrated in CSOM that neutrophil recruitment is increased to the infection site, and neutrophils in the middle ear promote bacterial biofilm stability through their contribution of dsDNA to the biofilm matrix^19^. These data support that induction of NETs elicited by biofilm contribute to the matrix of the bacterial aggregate and pinpoint the host immune response as a key factor in the chronicity of Pseudomonal bacterial infections.

Neutrophils may alter protein expression on bacterial cell walls and induce quorum signaling, further promoting conditions that support chronic infection and antibiotic tolerance^20,21^. Continuous recruitment of neutrophils to the site of infection may contribute to chronic infection through biofilm formation and inflammation that damages the host^22^. There is evidence that neutrophil elastase represses *P. aeruginosa* flagella expression and is selective for the non-motile biofilm phenotype^23^. Neutrophil elastase has been shown to contribute to disease pathogenesis and is a well-established marker of chronic bacterial infection; elastase presence is more damaging in chronic infection contrasting to acute infection^24^. Immunomodulation for treating other bacterial diseases has been successfully reported, providing the basis for a potential therapeutic solution^25,26^. Neutrophil immunomodulation has also been reported for other inflammation-mediated pathology such as atherosclerosis^27^. These lines of evidence suggest that adjunctive inhibition of elastase and other host-directed therapeutics might help counter the growing problem of ineffective antibiotics and promote clearance of *P. aeruginosa* biofilm infection. However, a potential role immunomodulation in the treatment of chronic middle ear bacterial disease, particularly CSOM, has not been explored.

Building off the foundation of many previously published animal models of acute and chronic otitis media that established microbial and host determinants of chronic infection^28–32^, we validated an animal model for CSOM^33^. In that model, we uncovered that chronic infection was dependent on dose, phenotype, and the proportion of *P. aeruginosa* persister cells, and demonstrated the ability of *P. aeruginosa* to form biofilm in vivo within 24 h^33^.

Here, using this model we investigated immunological changes that occur during the development of CSOM. We used machine learning to identify neutrophils as immunological drivers of *P. aeruginosa* CSOM in mice. Building on this result, we then tested the neutrophil elastase inhibitor GW 311616A in combination with ofloxacin as a potential adjuvant treatment regimen for *P. aeruginosa* CSOM. We further explored the potential of extracellular DNA as a biomarker for treatment response in CSOM and assessed the abundance of dsDNA in effusion from mice and human CSOM.

## Results

### Alterations in myeloid infiltrate at the site of infection in response to middle ear biofilm infection

Chronic infection models provide the opportunity to explore changes in cell response both as the infection initially establishes within the host and as the disease becomes chronic. Here, using our recently described CSOM mouse model, we assessed the host response to middle ear infection over time by multiparameter flow cytometry (FCM) analysis.

To this end, we created tympanic membrane perforations and occluded Eustachian tubes in two groups of mice. One day later, half the mice received 1.6 × 10^7^ CFU of *P. aeruginosa* PAO1 (PAO1.lux or PAO1.pUCP.eGFP) into the middle ear of C57Bl/6J mice via the ear canal. Control mice received an equal volume of sterile PBS. Bacterial density in the middle ear was measured by IVIS and by bacterial enumeration at the end of the experiment to confirm a chronic infection. We then collected 1–2 microliters of middle ear fluid from infected and control mice at 1, 14, and 28 days post-inoculation (d.p.i.) for FCM analysis.

**Fig. 1:**
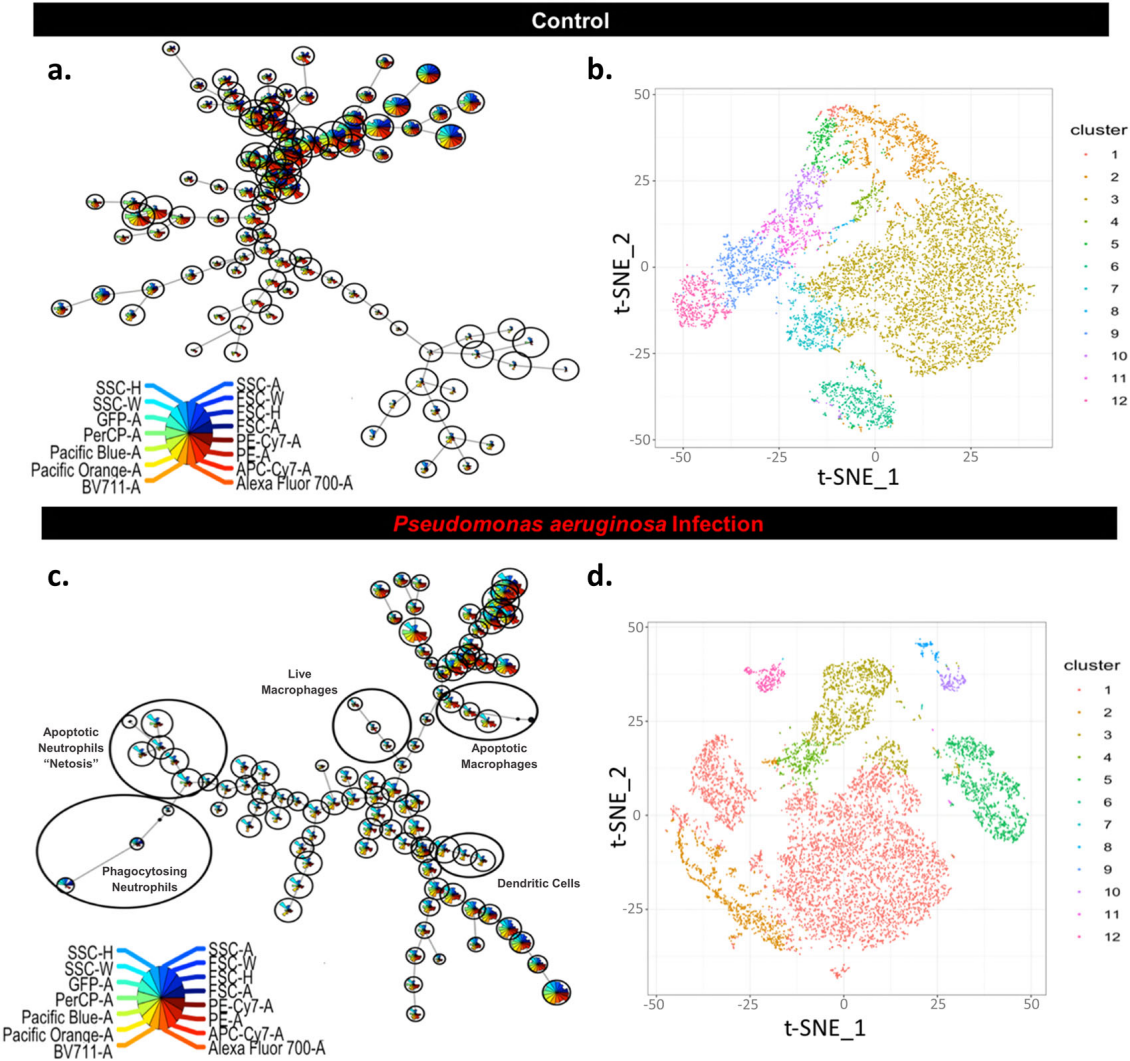
Assessment of myeloid clustering in chronic infection. The mouse CSOM middle ear effusion was assessed at 14 d.p.i. by FCM. Top panels: analysis of control mice. Bottom panels: analysis of infected mice. Tree-structured visualization of clusters by minimal spanning tree (MST), with cluster sizes correlating with the number of cells within the cluster (**a** control; **c** infected). Each point in the tree indicates single populations according to fluorescence intensity, and comparison of subpopulations in effusion from infected c.f. control animals. We manually annotated subpopulations in (**c**) for visual clarity. Minimal spanning tree diagram shows dimensionality reduction of cellular infiltrate in CSOM mice at 14 d.p.i. Representative MS trees of one uninfected (**a**) and one CSOM (**c**) sample. (*n* = 3–12 mice per group). t-SNE maps at 14 d.p.i. are shown with each color representing different subpopulations (1–12) based on scatter and fluorescent intensity (**b** control; **d** infected). Region size of the colored subpopulations is representative of increased cell density for the populations.

Noticeable shifts in myeloid populations were observed (Fig. 1). Both minimal spanning tree (MST) plots and t-SNE observations were indicative of neutrophil expansion and subpopulations changing in Ly6G expression. Neutrophils initially expanded within 24 h in infected and control mice, likely due to injury-associated inflammation from model creation (Supplementary Fig. 1A, B). In uninfected control mice, by the 2-week time point contraction occurred. In contrast, in infected mice we found that neutrophils remained the most abundant population. We also observed changes in neutrophil subpopulations over the course of infection. Bacteria-infected specimens maintained significant neutrophil populations over time and in the infected mice markers of cell death were extensively noted throughout multiple subpopulations (Supplementary Fig. 1C, D).

### Machine learning of infection response confirmed a potential immunomodulatory target

In order to improve interpretation of the FCM data, we used a machine learning approach. The FCM data were inserted into a binomial generalized linear model to computationally produce the maximum-likelihood target of infection status^34^.

Ly6G (Lymphocyte antigen complex 6 locus G6D), a specific surface marker of mature mouse neutrophils, was found to be a leading driver of infection in the middle ear 2 weeks post infection (Supplementary Fig. 2). The value of the coefficient of PE (−0.760013), including its *p* value (2e−16) and large absolute *z* value (−29.541) relative to other predictors, suggests a key role in influencing the infection status^34^. This information corroborated the subsets observed using t-SNE and MST, and further implicated a key role for neutrophils within the mouse model of CSOM.

**Fig. 2:**
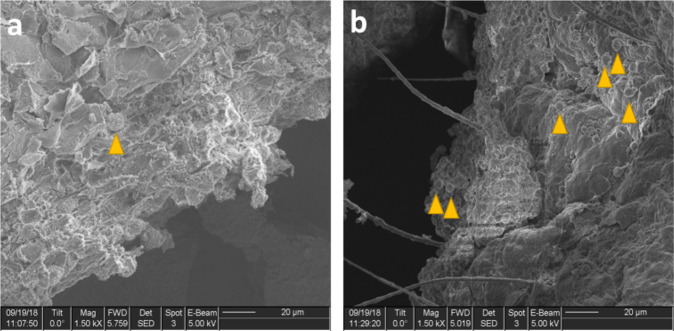
Scanning electron microscopy (SEM) images of extracted mucosa from the middle ear of a mouse model of *P. aeruginosa* CSOM. SEM images of extracted middle ear tissue. Neutrophils crowd mucosal surface after a biofilm begins to grow following inoculation with *P. aeruginosa*. Orange arrows indicate putative neutrophils on the mucosal surface. **a** SEM image taken from control middle ear mucosal surface. **b** SEM image taken from mucosa 1 d.p.i with PAO1. This experiment was repeated twice with highly similar results. C57BL/6J mice were inoculated with *P. aeruginosa* PAO1 or PBS one day following Eustachian tube occlusion and acute tympanic membrane perforation. Bar, 20 µm.

Consistent with this role for neutrophils, in SEM imaging of mucosa from the middle ear we observed large numbers of neutrophils on middle ear mucosa of infected mice post infection. In contrast in the control animals, we identified a single putative neutrophil on otherwise normal appearing mucosa (Fig. 2). Together these data indicate that the response to infection in this model is associated with an abundance of neutrophils in both middle ear mucosa and effusion.

### Alterations in neutrophil subsets and dsDNA at the site of infection in response to middle ear biofilm infection

**Fig. 3:**
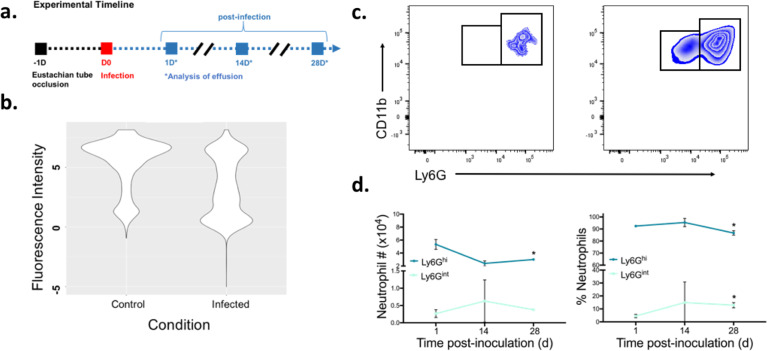
Neutrophil maturity diminishes over time in response to CSOM infection. Mice underwent trans-tympanic occlusion of the Eustachian tube 1 day prior to infection with PAO1. Neutrophils from middle ear effusion of *P. aeruginosa* infected CSOM mice were assessed by flow cytometry 1, 14, and 28 days post infection. **a** A timeline for the treatment and analysis steps used in this model. **b** Violin plot of Ly6G-PE fluorophore depicts a reduction in Ly6G in chronically infected CSOM mice, indicative of a reduction in neutrophil maturity. A representative plot from effusion of mice at 14 d.p.i. is shown. **c** Manual flow cytometry gating strategy for Ly6G^int^ (immature)/Ly6G^hi^ (mature). Representative plots for uninfected control (L) and CSOM (R) are shown at 14 d.p.i. **d** Neutrophil subpopulations were quantified by the gating strategy in (**c**) at 1, 14, and 28 d.p.i. Average of 3–5 biological replicates are shown. (*p < 0.05 by unpaired *t*-test; error bars show SD).

Using our CSOM mouse model, we examined neutrophil numbers and maturity in more detail over time in samples collected on days 1, 14, and 28 d.p.i. (Fig. 3a). We observed that neutrophils became phenotypically altered in Ly6G expression, experiencing a significant reduction in the LyG^hi^ subset by 14 d.p.i. (Fig. 3b). Next, we examined neutrophil subsets in the middle ear effusion of CSOM or control mice over time. Neutrophil subsets were manually gated based on Ly6G expression and CD11b+ CD45R− NK1.1− (Fig. 3c). Neutrophils remained the major cell type in effusion (>60%) post-inoculation. However, Ly6G^int^ (immature) neutrophils increased at the expense of the Ly6G^hi^ neutrophil subset in percent (approximately fivefold increase in Ly6G^int^ neutrophils, *p* < 0.05) and number (approximately fourfold increase in Ly6G^int^ neutrophils, *p* < 0.05) (Fig. 3d). These data demonstrate that with chronicity CSOM is associated with the accumulation of immature neutrophils in this model.

Given the abundance of neutrophils and the contribution of dead cells to extracellular DNA of bacterial biofilms^17,18^, we examined dsDNA levels resulting from CSOM infection in our model. We observed that dsDNA levels in CSOM were significantly greater compared to uninfected mice (mean of 4.5 ng/mL in uninfected control mice c.f. 32 ng/mL in CSOM mice, sevenfold change *p < 0.05) and increased over time (32–67 ng/mL, Supplementary Fig. 3). Together these data indicate that alterations in neutrophil subsets and dsDNA in middle ear effusion are associated with chronic biofilm infection of this model.

### Efficacy of GW 311616A combined with ofloxacin in CSOM

**Fig. 4:**
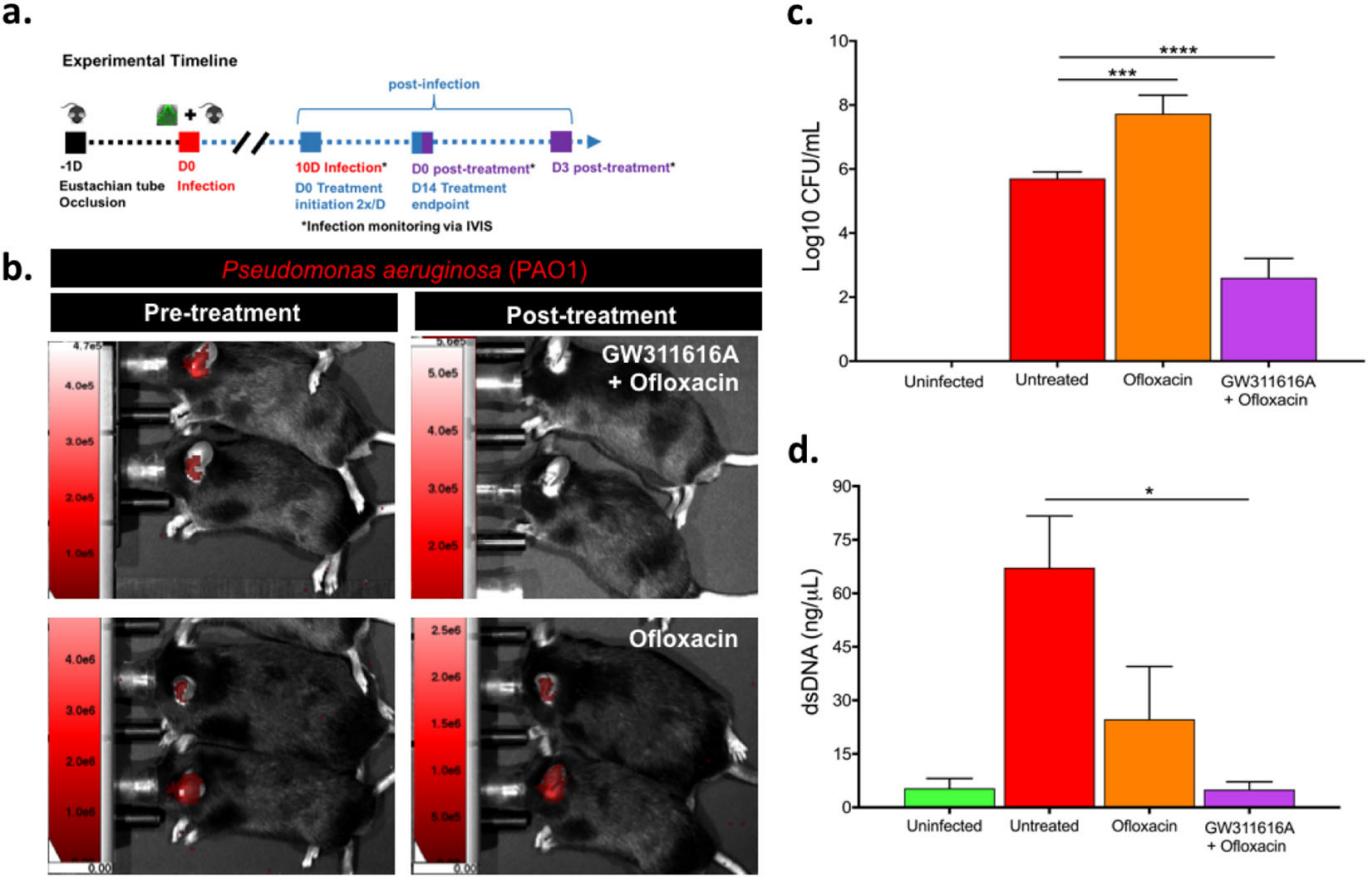
GW311616A plus ofloxacin shows efficacy for treating CSOM. Evaluation of treatment response by IVIS, CFU, and dsDNA following cessation of therapy. **a** Timeline for generation of a mouse model of *P. aeruginosa* CSOM. Mice were infected with PAO1.lux or PBS 1 d post-occlusion procedure (trans-tympanic occlusion of the Eustachian tube). After 10 d, chronic infection was confirmed and treatment initiated. Treatment was performed twice per day for 14 d and bacterial burden was assessed by IVIS and CFU after treatment recovery. **b** IVIS image of PAO1.lux CSOM mice post-treatment recovery following GW311616A combined with ofloxacin compared to ofloxacin alone on day 3 post-therapy cessation. Ofloxacin-only treated animals show return of infection compared to animals that received GW311616A combined with ofloxacin which showed no detectable recovery of infection. Untreated animals (*n* = 9) had consistent infection throughout the treatment window. **c** CFU/mL measured post-therapy cessation. GW311616A with ofloxacin significantly reduces bacterial burden relative to untreated animals (~3-log reduction, ****p < 0.0001 by ANOVA with Tukey–Kramer test; error bars show SEM). **d** dsDNA quantification in middle ear effusion of CSOM mice 3 days after cessation of treatment. GW311616A plus ofloxacin significantly reduced dsDNA levels relative to untreated CSOM infection (*p < 0.05). Ofloxacin alone was not significantly different than control mice. (*p < 0.05 by ANOVA with Tukey–Kramer test; error bars show SEM). (*n* = 3–12 mice per group).

The accumulation of an immature neutrophil population over time led us to investigate the role of neutrophils in treatment in this model. In conjunction with the fluoroquinolone antibiotic ofloxacin, the current standard of care for CSOM in humans, we treated mice with GW 311616A, a neutrophil elastase inhibitor and examined the impact of these regimens on bacterial load via IVIS and bacterial enumeration. A schematic of our protocol is provided in Fig. 4a.

Treatment with the neutrophil cytokine inhibitor GW 311616A plus ofloxacin led to non-detectable IVIS signal in mice treated with the immunomodulatory agent by the final day of treatment (day 14, Fig. 4b). A ~3-log reduction in bacterial burden was observed in excised homogenate of the infection site 3 days after treatment cessation (Fig. 4c). The PAO1 strain used in this study had a minimal inhibitory concentration (MIC) equal to 1.6 µg/ml for ofloxacin yet was unable to resolve chronic biofilm infection in vivo.

Using dsDNA as a biological marker of treatment, we likewise found the clinical response to GW 311616A combined with ofloxacin reduced levels of dsDNA to untreated (*p* > 0.05), bringing the response down to that of uninfected control levels. Treatment with ofloxacin alone (3 mg/ml) did not result in a significant difference between untreated mice (mean of 67 ng/mL in untreated c.f. mean of 25 ng/mL in ofloxacin only) (Fig. 4d). Together these data indicate that GW 311616A plus ofloxacin reduce bacterial burden and dsDNA in middle ear effusion in this model of Pseudomonal CSOM.

### Levels of dsDNA are increased in human CSOM

Given these observations in the animal model, we postulated that human clinical specimens might also have observable dsDNA levels. We therefore collected middle ear fluid from non-CSOM ears (*n* = 4, including cochlear implant surgery and dry tympanic membrane perforation repair) and middle ear fluid from ears of patients with CSOM (*n* = 5).

**Fig. 5:**
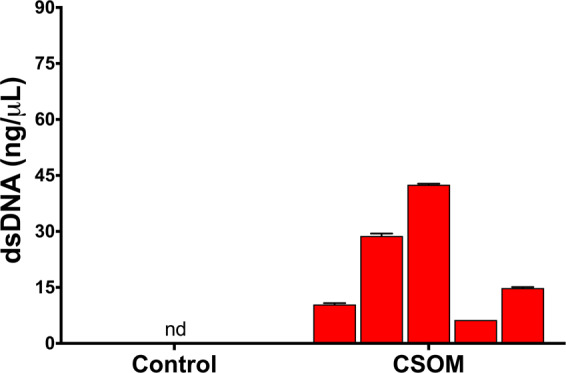
dsDNA is only present in the effusion of patient CSOM. Quantification of dsDNA in middle ear effusion supernatant in response to bacterial CSOM. Values for dsDNA from bacterial CSOM range from 6.3–42.5 ng/µL. Average value is increased versus control effusion supernatant (*n* = 4, patients required middle ear surgery for indication unrelated to bacteria). nd non-detectable.

We observed that middle ear fluid collected from CSOM patients (*n* = 5) had significantly increased dsDNA levels versus controls (range 6.3–42.5 ng/µL, *p < 0.05) (Fig. 5). In contrast, patients with conditions other than CSOM (control, *n* = 4) had dsDNA levels below the limit of detection. These data indicate that human CSOM is associated with elevated dsDNA levels in the middle ear and support the relevance of our animal model to human disease.

## Discussion

These results strongly implicate neutrophils in the ineffective immune response to *P. aeruginosa* infection observed in CSOM. We demonstrate increased numbers of neutrophils in a robust, recently described mouse model of CSOM as well as heightened dsDNA levels in human cases of the disease.

Moreover, our data indicate that it may be possible to target neutrophil elastase as an adjunctive treatment in CSOM. In functional studies in our newly established mouse model of CSOM, we demonstrated that treatment with neutrophil functional inhibitor potentiated ofloxacin treatment and resulted in a sustained reduction of bacterial burden greater than what is seen with ofloxacin alone. These data build on previous work of other biofilm infections that elastase-producing neutrophils at the site of infection may be contributing to the pathogenicity of *P. aeruginosa*. It is likely that elastase has additional function in promoting inflammation beyond tissue damage. In our model of CSOM, elastase inhibition potently synergizes with the antibiotic in clearance of the infection. It may engage both a direct effect on neutrophils and also collateral decrease in tissue inflammation. Interestingly, in a recent paper the authors found that elastase expression was the highest in immature neutrophils and is downregulated in mature neutrophils^35^. We found that CSOM is characterized by the shift in neutrophil phenotype to a more immature one. The mechanism of how exactly elastase inhibitor helps prevent the inflammation and infection is the subject of our further studies but we speculate that inhibiting elastase activity in immature neutrophils helps reduce DNA release and alters bacterial phenotype to render the bacterial cells more susceptible to antibiotic. Taken together, these data and our results encourage further studies of immunomodulatory agents against *P. aeruginosa* CSOM.

Bacteria find many ways to subvert host immunity to facilitate chronic infection. Indeed, multiple strategies from the bacteria to manipulate the host immune response have been reported^36,37^. Our study identified neutrophil Ly6G expression as an immunologic marker of CSOM infection. Ly6G is generally correlated with neutrophil differentiation and maturation and has been shown to be important for efficient anti-bacterial response^38^. Intriguingly, over the course of CSOM, Ly6G expression became very dim, meaning that over the course of infection, more immature neutrophils rather than mature neutrophils were recruited to the site. Bacterial infections can stimulate early neutrophil release from the bone marrow via CXCR4 signaling^39^. Similar phenotypic response is observed as part of emergency granulopoiesis in attempts to replace exhausted immune cells^40^. Importantly, phagocytosis and ROS production are higher in mature neutrophils and than immature ones, lending additional credence to the idea that rather than mounting an effective immune response these immature neutrophils may be feeding and protecting the biofilm, altering bacterial phenotype toward antibiotic tolerance^35^. Biofilm have taken advantage of the host immune response to facilitate survival in the hostile territory of the host and NET response in chronic infection is largely directed toward enhancing biofilm generation and bacterial phenotype alterations.

Our data support previous indications that dsDNA has value as a biomarker of CSOM treatment response^41,42^. There is great interest in identifying biomarkers for outcome prediction, especially in recurrent diseases in which assessment of a microbial cure remains limited^43^. The Food and Drug Administration has currently approved only eight biomarkers qualified for use in safety, diagnostic/prognostic, and monitoring of disease as part of the drug development process. We propose to use dsDNA as a biomarker to evaluate individual treatment response in the absence of a reproducible microbiological endpoint. This discovery allows infection status tracking via dsDNA quantitation from middle ear effusion.

We previously showed, in our animal model, that the conversion to inactive disease is likely temporary and this short term period is before recovery of the active disease. The Pseudomonas isolates used in this study are susceptible with MICs well within clinically relevant fluoroquinolone concentrations^44^. This supports that antibiotic resistance is not the reason for failure in our CSOM model. The likely mechanism, and likely underlying driving force in many recalcitrant infections such as CSOM, is the presence of persister cells within biofilm^45^. Persister cells maintain low metabolic activity and are less susceptible to antibiotic killing and their delayed growth resumption, allowing more time to recover from injury; further, after antibiotic cessation, persister cells resuscitate to become metabolically active. As persister cells are a likely candidate for recalcitrance in CSOM, a different choice of antibiotic, other than ofloxacin, is unlikely to improve overall efficacy.

Targeting the host immune system may serve as an effective adjunctive strategy for chronic infections caused by other antibiotic tolerant biofilm-associated pathogens. Particularly in diseases that allow for topical rather than systemic treatments, drug development of site-specific or topical immunosuppressant may be a more effective therapy as immediate mutational or stochastic bacterial responses are avoided. Consequently, site-specific inhibition of biofilm-associated neutrophils may represent a therapeutic route for treating chronic middle ear biofilm-associated infections. Therapeutic discovery combined with immunomodulation to permissive host features may provide an alternative approach toward disease eradication for other chronic biofilm infections. These data support the use of animal models of CSOM to identify targetable host mechanisms that can serve as potential drug target. While future discovery efforts are needed to further define the underlying pathogenesis in regard to immune-mediated infection maintenance, the methodology presented herein can be used to enhance early drug regime optimization for chronic biofilm infections.

## Methods

### Ethics statement

All human samples were collected with approval from the Stanford School of Medicine Institutional Review Board. Human collection was procured with informed consent for middle ear fluid from patient’s undergoing surgery for CSOM or non-CSOM conditions (cochlear implant surgery and dry tympanic membrane perforation repair). Animal procedures were approved by the Institutional Animal Care and Use Committee at Stanford University. Mice (2–6 months old C57BL/6J male and female) were purchased from Jackson Laboratory or bred and housed in the Stanford University vivarium with ad libitum access to food and water. Mice with vestibular symptoms received subcutaneous injections of sterile saline weekly (Cat. No. 0409-4888-02, Hospira, Lake Forest, IL, USA). Experiments were performed independently at least twice with 3–5 animals per group.

### Bacteria strains and preparation

*P. aeruginosa* PAO1 with constitutive expression of a chromosomal-encoded luminescence reporter (PAO1.lux) was constructed as previously described^46^. To create the fluorescent-tagged PAO1, a plasmid-encoded eGFP reporter (pUCP23.eGFP) was transformed into electrocompetent *P. aeruginosa* PAO1 (PAO1.pUCP23.eGFP) as previously described^46^. The expression of eGFP was confirmed via measurements at 478/510 nm. All organisms were cultured in LB broth from individual colonies at 37 °C, shaking at 200 RPM. Growth was monitored using a spectrophotometer at an optical density of 600 nm (OD_600_). For stationary cultures, bacteria were grown overnight and diluted to an OD of 0.8; the concentration used for inoculation (*P. aeruginosa* inoculum was 1.6 × 10^7^ CFU).

### CSOM model

We used a validated model of CSOM^33,47^. In brief, a sub-total tympanic membrane perforation and trans-tympanic Eustachian tube occlusion were performed 1 day prior to bacterial inoculation under anesthesia intraperitoneal injection of ketamine (65 mg/kg) and xylazine (5 mg/kg). The following day, model generation utilized direct inoculation of 10 µL bacterial inocula (1.6 × 10^7^ CFU) of luminescent or fluorescent *P. aeruginosa* PAO1 (PAO1.lux or PAO1.pUCP.eGFP) into the middle ear of C57Bl/6J mice via the ear canal under anesthesia of intraperitoneal injection of ketamine (65 mg/kg) and xylazine (5 mg/kg) or 3% isoflurane. Control mice received an equal volume of sterile PBS. Bacterial density in the middle ear was measured by IVIS and by bacterial enumeration at the end of the experiment to confirm a chronic infection. Groups of mice were euthanized after 1,14, and 28 days after infection whereupon tissues and middle ear fluid were collected.

### Real-time infection tracking

Disease progression was followed by capturing images with open emission using a LagoX In Vivo Imaging System (IVIS, Spectral Instruments Imaging, AZ, USA). Luminescence was quantified with the Aura software (Spectral Instruments Imaging, AZ, USA). Briefly, mice were placed on the right lateral position to expose the left ear at progressive days post-inoculation. Images were initially acquired at 60 s exposure with medium binning. If no signal was detected, mice were reanalyzed at 300 s exposure with high binning. Background luminescent signal was subtracted from signal coming from the area around the ear. Chronic infection was designated as the presence of infection 10 days post inoculation.

### Antibiotics and immunomodulatory treatment

The neutrophil elastase inhibitor GW 311616A (2 mg/ml) (Axon Medchem, Groningen, Netherlands) was used in combination with 3% (3 mg/ml) ofloxacin Otic Solution (Apotex Corp, Weston, FL, USA) by dissolving in the antibiotic solution. Antibiotic or antibiotic-inhibitor combination (10 µL) were directly inoculated into the middle ear through an opened tympanic membrane wound generated as described in model generation. Antibiotic treatment alone or antibiotic with GW 311616A was performed twice daily for 2 weeks, allowing the mouse to lie on the ventral side for 5 min post treatment and then recover from anesthesia (2% isoflurane). Progression was monitored by IVIS as described above.

### Broth microdilution for the minimum inhibitory concentration (MIC)

The MIC of the ofloxacin was determined against PAO1 using the broth microdilution method. The bacteria were grown overnight at 37 °C in LB medium. The drug was mixed with bacterial inoculum in LB (optical density OD at 600; OD600 = 0.2) and serial diluted (twofold) in 96-well polypropylene microplates. After overnight incubation, bacteria growth in the presence of the various drug concentrations was evaluated by visual observation of the solution (clear or cloudy) in the wells. The MIC was obtained from the lowest concentration of the drug, which show no bacterial growth.

### Flow cytometry

Monoclonal antibodies, labeled with appropriate fluorochromes were purchased from BD Biosciences (San Jose, CA, USA), Invitrogen (Carlsbad, CA, USA), or Life Technologies (Carlsbad, CA USA). Cells isolated from the middle ear effusion were stained for Ly6G (clone 1A8), Ly6C (clone AL-21), NK1.1 (clone PK136), CD45R (B220 clone RA3-6B2), CD11c (clone HL3) (BD Biosciences, San Jose, CA, USA), CD11b (clone M1/70) (Invitrogen, Carlsbad, CA, USA), and live/dead markers, including nucleic acid Sytox dyes and amine-reactive dyes (Life Technologies, Carlsbad, CA USA). This allowed discrimination of neutrophil subsets based on staining of Ly6G: CD11b+ CD45R− NK1.1− Ly6G^hi^ mature cells; CD11b+ CD45R− NK1.1− Ly6G^int^ immature cells. Macrophages were identified as CD11b± CD45R− NK1.1− Ly6G− Ly6C+; and dendritic cells as CD11c+^48^. Cells undergoing phagocytosis were identified as positive for surface markers of subsets as above and also positive for the bacterial reporter GFP. Cells were analyzed on an LSRII flow cytometer (BD Biosciences, San Jose, CA, USA) and analysis was performed using FlowJo (BD Biosciences, San Jose, CA, USA). All samples were analyzed the same day as the samples were collected and cell identification by sequential gating performed on live cells with doublet discrimination.

### Bioinformatics analysis

Changes in myeloid infiltrate due to infection were visualized utilizing MST diagrams, a method used to assess how populations are related as well as determine the respective sizes of each population. We further screened subpopulation changes using t-SNE projections with FlowSOM clustering to visualize relation and abundance of subpopulations^49–51^. t-SNE maps are shown with a single color representing subpopulations (1–12) based on scatter and fluorescent intensity as measured by FCM. Region size is representative of increased cell density for the populations. Myeloid immunophenotype markers were also mapped by MST, with each point in the tree indicating single populations according to fluorescence intensity as measured by FCM. A plot is drawn in which each node is represented by a star chart indicating the median fluorescence intensities. Specifically, the tree plot shows median fluorescence intensity values of each protein marker. The FCM data were also inserted into a binomial generalized linear model to computationally discover the maximum-likelihood target of infection status^34^.

### Bacteria quantitation

The site of infection in the middle ear was harvested to determine bacterial density. To determine bacteria burden via serial dilution, the tissues were homogenized in 1 mL sterile PBS and incubated for >2 h at 4 °C with shaking. Subsequently, the suspension was diluted and drop plated on LB agar for bacterial enumeration. Replicates were performed within the countable range with every sample. The limit of detection is 10^2^ CFU/mL.

### Nucleic acid quantitation in murine CSOM

The AccuBlue Broad Range dsDNA Quantitation Kit (Cat. No. 31007, Biotium, Fremont, CA, USA) was used to detect dsDNA in each of the samples. Samples were blind-tested, and the concentration of dsDNA was calculated from the fluorescence readings of a microplate reader. Each plate contained middle ear effusion samples, dsDNA standards provided by the quantitation kit, a water control, and a PBS control. Sample were assessed in duplicate. Water and PBS controls served to correct from the background interference readings on the microplate reader.

This kit was used to quantify dsDNA in mouse samples at various time points after infection. Control mice and mice day 3 and day 7 post infection were used to compare the progression of the disease. The 3D control group had a sterile effusion sample collected at the same time as the infected sample. dsDNA concentration was calculated from microplate fluorescence readings.

### Nucleic acid quantitation in human CSOM

Informed consent was obtained from all human participants. Human samples of middle ear effusion of patients undergoing surgery for CSOM and conditions unrelated to bacterial infections were collected and stored in liquid nitrogen until analysis. Using the AccuBlue Broad Range dsDNA Quantitation Kit (Cat. No. 31007, Biotium, Fremont, CA USA) as above, positive samples were matched with patients with known CSOM infections, while the negative samples were matched the patients with no infection (e.g., cochlea implant surgery negative controls). Analysts were double-blinded to the status of the patient.

### Scanning electron microscopy and sample processing

Middle ear samples were fixed in 2% glutaraldehyde/4% formaldehyde in sodium cacodylate buffer (pH 7.3) for 24 h at room temperature and then stored at 4 °C. 1% OsO4 was added followed by sequential ethanol washes of 50%, 70%, 95%, and 100%. To remove the ethanol and dry the sample, HMDS was used and then allowed to air dry. The Critical Point Dryer (CPD) was used to remove the remaining ethanol. Sample was then attached to a 12 mm stub using double stick, carbon-conductive tape and coated with gold/paladium (Au/Pd) at a 60:40 ratio. Using a FEI Strata 235DB dual-beam scanning electron microscopy (FIB-SEM), images of *P. aeruginosa* infected middle ear mucosa were taken.

### Statistical analysis

Analysis was performed using GraphPad Prism 7.0 or 8.0 (GraphPad Software, Inc., La Jolla, CA) to test statistical significance. *P* values were calculated using either unpaired *t*-test; or one-way ANOVA with Tukey–Kramer test. *P* values <0.05 were considered significant.

### Reporting summary

Further information on research design is available in the Nature Research Reporting Summary linked to this article.

## Supplementary information

Supplementary Information

Reporting Summary

## Data Availability

All data needed to evaluate the conclusions in the paper are present in the paper. Additional data related to this paper may be requested from the corresponding author.
