# Supplementary material for: Treatment with a neutrophil elastase inhibitor and ofloxacin reduces P. aeruginosa burden in a mouse model of chronic suppurative otitis media
Source: NPJ Biofilms Microbiomes. 2021 Apr 6;7:31. doi: 10.1038/s41522-021-00200-z (PMC8024339; doi:10.1038/s41522-021-00200-z)
Supplement: Supplementary file 1 — Supplementary Information [file 41522_2021_200_MOESM1_ESM.pdf]

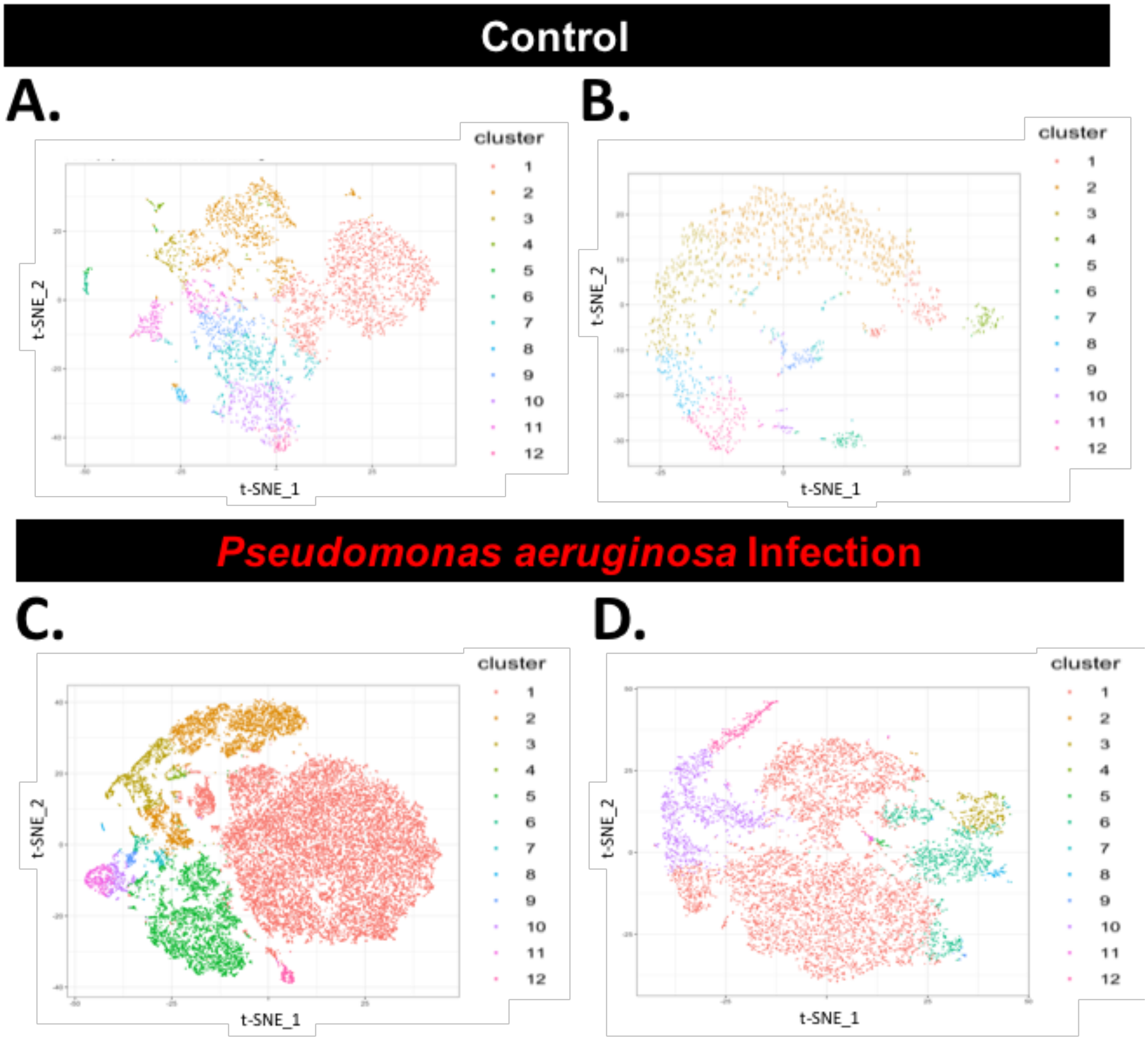

Supplementary Figure 1: Myeloid clustering in response to chronic infection. Middle ear effusion from mice with CSOM or control were assessed over time (1 d.p.i., 28 d.p.i.) by FCM. Representative t-SNE maps of uninfected (top) and CSOM (bottom) samples compare subpopulations in effusion from infected and control animals (n = 3 - 12 mice per group). Top panels: Analysis of control mice (A, control 1 d.p.i.; B, control 28 d.p.i.). Bottom panels: Analysis of infected mice (C, infected 1 d.p.i.; D, infected 28 d.p.i.). t-SNE maps are shown with a single color representing subpopulations (1-12) based on scatter and fluorescent intensity. Region size is representative of increased cell density for a population.

```
Call:
glm(formula = Infection.status ~ `FSC-A` + `FSC-H` + `FSC-W` +
    `SSC-A` + `SSC-H` + `SSC-W` + `GFP-A` + `PerCP-A` + `Pacific Blue-A` +
    `Pacific Orange-A` + `BV711-A` + `Alexa Fluor 700-A` + `APC-Cy7-A` +
    `PE-A` + `PE-Cy7-A`, family = binomial, data = myInput)

Deviance Residuals:
    Min       1Q   Median       3Q      Max
-3.6236  -0.8427  -0.1355   0.8312   2.9641

Coefficients:
                     Estimate Std. Error z value Pr(>|z|)
(Intercept)         28.503981   2.628931  10.842  < 2e-16 ***
`FSC-A`              1.029223   0.230936   4.457 8.32e-06 ***
`FSC-H`             -1.166250   0.232394  -5.018 5.21e-07 ***
`FSC-W`             -3.077122   0.341645  -9.007  < 2e-16 ***
`SSC-A`             -0.220269   0.209541  -1.051  0.29317
`SSC-H`              0.406238   0.212529   1.911  0.05595 .
`SSC-W`             -0.852401   0.199592  -4.271 1.95e-05 ***
`GFP-A`              0.977082   0.036097  27.068  < 2e-16 ***
`PerCP-A`           -0.009236   0.023728  -0.389  0.69710
`Pacific Blue-A`     0.167678   0.024283   6.905 5.01e-12 ***
`Pacific Orange-A`  -0.021703   0.016753  -1.295  0.19515
`BV711-A`            0.057140   0.020439   2.796  0.00518 **
`Alexa Fluor 700-A`  0.166987   0.022237   7.509 5.94e-14 ***
`APC-Cy7-A`         -0.058686   0.018807  -3.120  0.00181 **
`PE-A`              -0.760013   0.025728 -29.541  < 2e-16 ***
`PE-Cy7-A`          -0.082104   0.029074  -2.824  0.00474 **
---
Signif. codes:  0 ‘***’ 0.001 ‘**’ 0.01 ‘*’ 0.05 ‘.’ 0.1 ‘ ’ 1

(Dispersion parameter for binomial family taken to be 1)

    Null deviance: 24909  on 17967  degrees of freedom
Residual deviance: 19945  on 17952  degrees of freedom
AIC: 19977

Number of Fisher Scoring iterations: 7
```

Supplementary Figure 2: Binomial generalized linear model (GLM) with infection status as the dependent variable.

The value of the coefficient of PE (Ly6G) (-0.760013), including its p-value (2e-16) and large absolute z value (-29.541, highlighted in white box) relative to other predictors, suggests that it plays a key role in influencing the infection status.

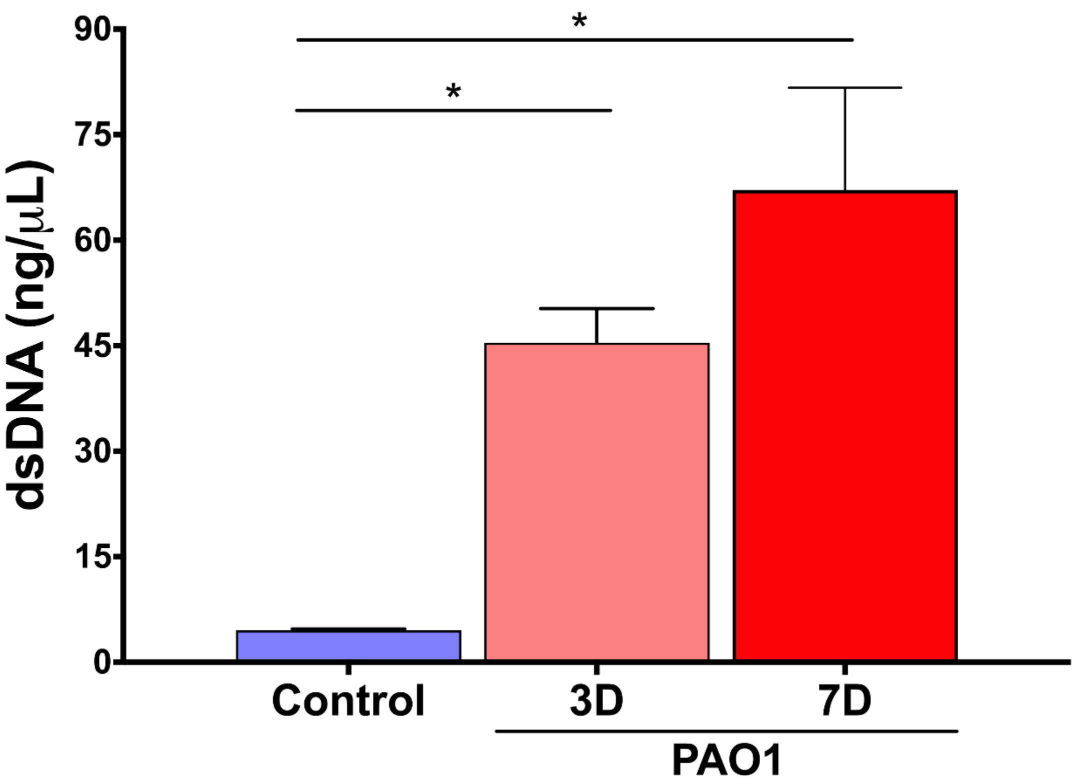

Supplementary Figure 3: CSOM increases dsDNA at the site of infection. Quantification of dsDNA from middle ear effusion in CSOM. The presence of dsDNA was observed in effusion from *P. aeruginosa* infected mice with CSOM. Elevated levels of dsDNA were observed in infected mice (light and dark red bars) compared to uninfected control mice (sterile effusion taken 3 d after Eustachian tube occlusion and PBS vehicle inoculation, blue column). Results are for biological replicates of 3-5 mice per group. (*$p<0.05$ by unpaired t test; error bars show SEM).
