# Supplementary material for: Treatment with a neutrophil elastase inhibitor and ofloxacin reduces P. aeruginosa burden in a mouse model of chronic suppurative otitis media
Source: NPJ Biofilms Microbiomes. 2021 Apr 6;7:31. doi: 10.1038/s41522-021-00200-z (PMC8024339; doi:10.1038/s41522-021-00200-z)
Supplement: Supplementary file 2 — Reporting Summary [file 41522_2021_200_MOESM2_ESM.pdf]

nature research

Corresponding author(s): Khomtchouk et al.

Last updated by author(s): Dec 20, 2020

Reporting Summary

Nature Research wishes to improve the reproducibility of the work that we publish. This form provides structure for consistency and transparency in reporting. For further information on Nature Research policies, see our Editorial Policies and the Editorial Policy Checklist.

Statistics

For all statistical analyses, confirm that the following items are present in the figure legend, table legend, main text, or Methods section.

n/a	Confirmed	
☐	☒	The exact sample size (*n*) for each experimental group/condition, given as a discrete number and unit of measurement
☒	☐	A statement on whether measurements were taken from distinct samples or whether the same sample was measured repeatedly
☐	☒	The statistical test(s) used AND whether they are one- or two-sided<br>*Only common tests should be described solely by name; describe more complex techniques in the Methods section.*
☒	☐	A description of all covariates tested
☒	☐	A description of any assumptions or corrections, such as tests of normality and adjustment for multiple comparisons
☐	☒	A full description of the statistical parameters including central tendency (e.g. means) or other basic estimates (e.g. regression coefficient) AND variation (e.g. standard deviation) or associated estimates of uncertainty (e.g. confidence intervals)
☐	☒	For null hypothesis testing, the test statistic (e.g. *F*, *t*, *r*) with confidence intervals, effect sizes, degrees of freedom and *P* value noted<br>*Give P values as exact values whenever suitable.*
☒	☐	For Bayesian analysis, information on the choice of priors and Markov chain Monte Carlo settings
☒	☐	For hierarchical and complex designs, identification of the appropriate level for tests and full reporting of outcomes
☒	☐	Estimates of effect sizes (e.g. Cohen's *d*, Pearson's *r*), indicating how they were calculated

Our web collection on statistics for biologists contains articles on many of the points above.

Software and code

Policy information about availability of computer code

Data collection: *Provide a description of all commercial, open source and custom code used to collect the data in this study, specifying the version used OR state that no software was used.*

Data analysis: *Provide a description of all commercial, open source and custom code used to analyse the data in this study, specifying the version used OR state that no software was used.*

For manuscripts utilizing custom algorithms or software that are central to the research but not yet described in published literature, software must be made available to editors and reviewers. We strongly encourage code deposition in a community repository (e.g. GitHub). See the Nature Research guidelines for submitting code & software for further information.

Data

Policy information about availability of data

All manuscripts must include a data availability statement. This statement should provide the following information, where applicable:

- Accession codes, unique identifiers, or web links for publicly available datasets
- A list of figures that have associated raw data
- A description of any restrictions on data availability

All data needed to evaluate the conclusions in the paper are present in the paper. Additional data related to this paper may be requested from the authors.

Field-specific reporting

Please select the one below that is the best fit for your research. If you are not sure, read the appropriate sections before making your selection.

☒ Life sciences ☐ Behavioural & social sciences ☐ Ecological, evolutionary & environmental sciences

For a reference copy of the document with all sections, see nature.com/documents/nr-reporting-summary-flat.pdf

Life sciences study design

All studies must disclose on these points even when the disclosure is negative.

Sample size	*Describe how sample size was determined, detailing any statistical methods used to predetermine sample size OR if no sample-size calculation was performed, describe how sample sizes were chosen and provide a rationale for why these sample sizes are sufficient.*
Data exclusions	*Describe any data exclusions. If no data were excluded from the analyses, state so OR if data were excluded, describe the exclusions and the rationale behind them, indicating whether exclusion criteria were pre-established.*
Replication	*Describe the measures taken to verify the reproducibility of the experimental findings. If all attempts at replication were successful, confirm this OR if there are any findings that were not replicated or cannot be reproduced, note this and describe why.*
Randomization	*Describe how samples/organisms/participants were allocated into experimental groups. If allocation was not random, describe how covariates were controlled OR if this is not relevant to your study, explain why.*
Blinding	*Describe whether the investigators were blinded to group allocation during data collection and/or analysis. If blinding was not possible, describe why OR explain why blinding was not relevant to your study.*

Reporting for specific materials, systems and methods

We require information from authors about some types of materials, experimental systems and methods used in many studies. Here, indicate whether each material, system or method listed is relevant to your study. If you are not sure if a list item applies to your research, read the appropriate section before selecting a response.

Materials & experimental systems

n/a	Involved in the study	
☐	☒	Antibodies
☒	☐	Eukaryotic cell lines
☒	☐	Palaeontology and archaeology
☐	☒	Animals and other organisms
☒	☐	Human research participants
☒	☐	Clinical data
☒	☐	Dual use research of concern

Methods

n/a	Involved in the study	
☒	☐	ChIP-seq
☐	☒	Flow cytometry
☒	☐	MRI-based neuroimaging

Antibodies

Antibodies used	Ly6G (clone 1A8), Ly6C (clone AL-21), NK1.1 (clone PK136), CD45R (B220 clone RA3-6B2), CD11c (clone HL3) (BD Biosciences, San Jose, CA USA), CD11b (clone M1/70) (Invitrogen, Carlsbad, CA USA), and live/dead markers, including nucleic acid Sytox dyes and amine-reactive dyes (Life Technologies, Carlsbad, CA USA)
Validation	BD believes antibody validation is critical to ensure accurate scientific results. The product development process includes testing on a combination of primary cells, cell lines and/or transfectant cell models with relevant controls using multiple immunoassays to ensure biological accuracy. We also perform multiplexing with additional antibodies to interrogate antibody staining in multiple cell populations. Invitrogen antibodies are currently undergoing a rigorous 2-part testing approach, incl. Part 1—Target specificity verification and Part 2—Functional application validation.

Animals and other organisms

Policy information about studies involving animals; ARRIVE guidelines recommended for reporting animal research

Laboratory animals	Mice used in the study were 2-6 months old C57BL/6J male and female.
Wild animals	The study did not involve wild animals.
Field-collected samples	The study did not involve samples collected from the field.

Ethics oversight | The Stanford University School of Medicine was responsible for study approval.

Note that full information on the approval of the study protocol must also be provided in the manuscript.

Flow Cytometry

Plots

Confirm that:

☐ The axis labels state the marker and fluorochrome used (e.g. CD4-FITC).

☒ The axis scales are clearly visible. Include numbers along axes only for bottom left plot of group (a 'group' is an analysis of identical markers).

☐ All plots are contour plots with outliers or pseudocolor plots.

☒ A numerical value for number of cells or percentage (with statistics) is provided.

Methodology

Sample preparation	All samples were analyzed the same day as the samples were collected. Cells were isolated from the middle ear effusion and stained as follows.
Instrument	Cells were analyzed on an LSRII flow cytometer (BD Biosciences, San Jose, CA USA).
Software	Analysis was performed using FlowJo (BD Biosciences, San Jose, CA USA).
Cell population abundance	Sorting was not performed.
Gating strategy	Cell identification was performed by sequential gating performed on live cells with doublet discrimination. Cells isolated from the middle ear effusion were stained for Ly6G (clone 1A8), Ly6C (clone AL-21), NK1.1 (clone PK136), CD45R (B220 clone RA3-6B2), CD11c (clone HL3) (BD Biosciences, San Jose, CA USA), CD11b (clone M1/70) (Invitrogen, Carlsbad, CA USA), and live/dead markers, including nucleic acid Sytox dyes and amine-reactive dyes (Life Technologies, Carlsbad, CA USA). This allowed discrimination of neutrophil subsets based on staining of Ly6G: CD11b+ CD45R− NK1.1− Ly6Ghi mature cells; CD11b+ CD45R− NK1.1− Ly6Gint immature cells. Macrophages were identified as CD11b+/- CD45R− NK1.1− Ly6G− Ly6C+; and dendritic cells as CD11c+ (Rose et al., 2012). Phagocytosing cells were identified as surface marker positive for subsets as above and also positive for the bacterial reporter GFP.

☐ Tick this box to confirm that a figure exemplifying the gating strategy is provided in the Supplementary Information.
